# IgE-Mediated Fish Allergy in Children

**DOI:** 10.3390/medicina57010076

**Published:** 2021-01-18

**Authors:** Betul Buyuktiryaki, Marzio Masini, Francesca Mori, Simona Barni, Giulia Liccioli, Lucrezia Sarti, Lorenzo Lodi, Mattia Giovannini, George du Toit, Andreas Ludwig Lopata, Maria Andreina Marques-Mejias

**Affiliations:** 1 Division of Pediatric Allergy, Koc University Hospital, 34010 Istanbul, Turkey; betulbuyuktiryaki@yahoo.com; 2 Department of Pediatrics, Sapienza University of Rome, 00185 Rome, Italy; marzio.masini@gmail.com; 3 Allergy Unit, Department of Pediatrics, Meyer Children’s University Hospital, 50139 Florence, Italy; francesca.mori@meyer.it (F.M.); simona.barni@meyer.it (S.B.); giulia.liccioli@meyer.it (G.L.); lucrezia.sarti@gmail.com (L.S.); 4 Department of Health Sciences, Division of Immunology, Section of Pediatrics, University of Florence and Meyer Children’s Hospital, 50139 Florence, Italy; lorenzo.lodi@unifi.it; 5 Pediatric Allergy Group, Department of Women and Children’s Health, School of Life Course Sciences, King’s College London, London SE5 9NU, UK; George.DuToit@gstt.nhs.uk (G.d.T.); AMarquesMejias@gstt.nhs.uk (M.A.M.-M.); 6 Children’s Allergy Service, Evelina London Children’s Hospital, Guy’s and St Thomas’ NHS Foundation Trust, London SE1 7EH, UK; 7 Peter Gorer Department of Immunobiology, School of Immunology & Microbial Sciences, King’s College London, London SE5 9NU, UK; 8 Molecular Allergy Research Laboratory, College of Public Health, Medical and Veterinary Sciences, Australian Institute of Tropical Health and Medicine, James Cook University, Townsville, QLD 4811, Australia; andreas.lopata@jcu.edu.au

**Keywords:** allergen, basophil activation test, component resolved diagnosis, fish allergy, immunoglobulin E, management, oral food challenge, parvalbumin, paediatrics, skin prick test

## Abstract

Fish allergy constitutes a severe problem worldwide. Its prevalence has been calculated as high as 7% in paediatric populations, and in many cases, it persists into adulthood with life-threatening signs and symptoms. The following review focuses on the epidemiology of Immunoglobulin E (IgE)-mediated fish allergy, its pathogenesis, clinical manifestations, and a thorough approach to diagnosis and management in the paediatric population. The traditional approach for managing fish allergy is avoidance and rescue medication for accidental exposures. Food avoidance poses many obstacles and is not easily maintained. In the specific case of fish, food is also not the only source of allergens; aerosolisation of fish proteins when cooking is a common source of highly allergenic parvalbumin, and elimination diets cannot prevent these contacts. Novel management approaches based on immunomodulation are a promising strategy for the future of these patients.

## 1. Introduction

Fish consumption has seen a steady rise in recent years, both in adults and children. Multiple factors play a part in this trend, including the benefits of an increased intake of antioxidants and omega-3 fatty acids, making fish meat of fundamental importance in the growing child’s diet. Furthermore, it has been proposed that increased representation of omega-3 fatty acids in a child’s diet could play a significant role in preventing future development of atopic diseases [1,2,3]. However, fish allergy can represent a severe problem worldwide, with a prevalence as high as 7% in the paediatric population [4], persisting in many cases into adulthood with life-threatening signs and symptoms.

This review aims to outline the epidemiology of Immunoglobulin E (IgE)-mediated fish allergy in children across the globe, its pathogenesis, clinical manifestations, and an in-depth approach to diagnosis, focusing on serological and molecular investigations. We will finally discuss traditional and novel approaches and attitudes towards fish allergy management and therapy and prospects for its future.

## 2. Epidemiology

Fish is one of the most common foods responsible for allergic reactions in children and adults worldwide [1,2,4]. Since only a minority of fish allergy cases tend to resolve with age [5,6], this clinical condition is routinely reported as having a higher prevalence in adults than in children, the opposite of what is usually observed in other common allergies of childhood, like dairy and egg. The exact rates of fish allergy are hard to quantify since multiple variable factors are involved in how prevalence is measured. In addition to the age mentioned above, other factors are critical, such as geography: fish allergy is more common in countries where fish is one of the staples in the culture’s diet, so fish consumption is higher. Another variable is the way allergy is assessed, e.g., self-reported, confirmed by a doctor, confirmed with skin prick test (SPT) or serum specific IgE (sIgE), or confirmed by oral food challenges (OFCs), with higher numbers observed with the first methods and lower with the latter ones.

When investigating with self/parent-assessed methods, the highest prevalence was measured in Finnish children, ranging from 5% [7] to 7% [8]. On the contrary, the lowest one (0.0001%) was measured in 0–2-year-olds in Israel [9]. A significant prevalence is reported in the United Arab Emirates [10], with 2.8% of children from 6 to 9 reported to have reacted to fish’s ingestion. In Europe, the highest prevalence was found in countries with traditionally fish-centric diets, including Finland as well as other Scandinavian countries such as Norway (3% [11]), and Spain [12]: in a study by Crespo et al. [13] fish was responsible for 17.8% of allergic reactions documented in a cohort of paediatric patients with SPT and sIgE-positive food allergy.

Indeed, those numbers progressively get lower when we consider studies where sensitisation was assessed with in-vivo or in-vitro methods: in Europe, Finnish children show a sensitisation incidence of 0.3% [14], higher rates have been reported for British (1.3% [15]) and French (0.7% [16]) children. Even fewer children were classified as fish-allergic when we consider studies where OFCs were performed: 0% in Denmark [17], 0.0006% in the United Kingdom [18], 0.0002% in Turkey [19], and 0.2% in Iceland [20]. The overall point-prevalence of OFC-confirmed fish allergy in Europe was estimated at 0.06% in a meta-analysis by Nwaru et al. [21].

Fish consumption is perhaps the highest in the Asian continent, with Japan averaging 54 kg yearly per capita, reflecting on the high prevalence rates of fish allergy observed in multiple Asian countries [22]. Connett et al. in 2012 reported a prevalence of 2.29%, 0.26%, and 0.29%, respectively, in the Philippines, Singapore, and Thailand by polling a cohort of 25,842 14–16-year old students [23]. Another study by Lao-Araya, using parent questionnaires, showed a lifetime prevalence in Thai preschoolers of 1.1% [24]. In a cross-sectional study in over 8000 children in Vietnam, aged 2–6 years, 1.62% had a self-reported allergy to fish, while the doctor-diagnosed one dropped to 1.24% of the population examined [25]. In China, a study conducted on infants reported a prevalence of fish allergy of 0.21%, confirmed by SPT [26], reflecting a difference in prevalence between self-reported studies and SPT-confirmed/doctor diagnosed studies.

In the American continent, the highest prevalence, as recorded with random telephone survey, was seen in the United States (US), where a study polled more than 38,000 children aged 0–18 with a resulting fish allergy prevalence ranging from 0.3% (0–2 years old) to 0.6% (>11 years old) [27]. A recent study showed a remarkable, dramatic difference in fish allergy prevalence in different ethnic groups in the US: in a cohort of sIgE- or SPT-confirmed food-allergic patients, fish was the culprit allergen in 3.4% of white patients, 16.16% of Hispanics, and an outstanding 34.39% of African American children, showing the overwhelming ethnic influence over simple geography when determining food sensitisation [28]. In Canadian children, the prevalence was 0.18% when considering “probable allergy” (compatible clinical history reported by parent questionnaires) [29].

African data is scarce: a 2010 questionnaire-based study showed a prevalence of fish allergy of 0.3% in Ghanaian children aged 5–16 [30].

## 3. Pathogenesis and Clinical Features

Adverse reactions to fish are widespread in adults and children, but IgE-mediated reactions represent only a fraction (albeit the most common). A summary of immunological and nonimmunological adverse reactions will be outlined.

### 3.1. Nonimmunological Adverse Reactions

- Anisakiasis: infection by the parasite Anisakis, resulting in mostly gastrointestinal clinical manifestations. It requires the ingestion of live parasites. Therefore it is only contracted after consuming raw, undercooked, or pickled fish [31].
- Scombroid poisoning: a syndrome caused by ingestion of poorly preserved fish (more often red meat fish like tuna), in which bacterial overgrowth allows histidine to be converted into histamine. Clinical manifestations mimic allergic reactions, with rapid onset (around 30 min after ingestion), for example, urticaria, oral allergic syndrome, nausea and vomiting, and, in rare cases, anaphylaxis. Patients, who often do not have a history of fish allergy, often report oral tingling sensation and metallic flavour when eating the responsible fish, and usually, the same signs and symptoms are reported by other family members who consumed the same food [32,33].
- Toxic algae poisoning: fish can consume several toxin-producing algae taken up by filter feeders such as mussels and clamps. Subsequently, the human ingestion of contaminated fish triggers this type of poisoning. The clinical manifestations are varied and depend on the toxin: e.g., Ciguatera, due to ciguatoxin found most commonly in tropical fishes (groupers, eel, Spanish mackerel), may present with cutaneous (urticaria), gastrointestinal (nausea, vomiting), neurological (blurred vision, paraesthesia, ataxia, seizures) and cardiovascular (bradycardia/tachycardia, hypotension/hypertension, conduction block) signs and symptoms [34,35].
- Bacterial/viral contamination: eating fish raised in or harvested from contaminated waters will result mostly in gastrointestinal clinical manifestations arising several hours after ingestion, often accompanied by fever [36].
- Seafood intolerance: due to vasoactive amines present in fish (histamine and tyramine), especially when canned or pickled, or fish autolysates [37]. Usually presents itself with a headache.

### 3.2. Immunological Adverse Reactions

- IgE-mediated adverse reactions to fish: the most common form of adverse reaction to fish, which involves the development of sensitisation, a type 2 T helper (Th2) response, and production of sIgEs against fish allergens. Its pathogenesis and clinical features are discussed in more detail below.
- Non-IgE-mediated adverse reactions to fish: they include Food Protein-Induced Enterocolitis Syndrome (FPIES) and Food Protein-Induced Allergic Proctocolitis (FPIAP), of which fish is a major causative agent [38,39,40], and eosinophilic esophagitis (EoE)/gastritis [41,42,43]. In the management of EoE, an empiric six-food elimination diet is generally recommended and includes the elimination of fish/shellfish along with milk, egg, wheat, nuts, and soy [41,42,43,44]. Of note, some clinicians advise a four-food elimination diet and allow taking nuts and fish.
- Immunological, IgE-mediated adverse reactions to parasite infested fish. An immunological, IgE-mediated adverse reaction to Anisakis could occur due to the sensitisation to the nematode’s proteins, which infests various fish species. The clinical presentation is indistinguishable from a fish allergy, but sIgEs are not directed towards fish protein but rather towards the parasite. Thus, SPT and sIgE will detect Anisakis sensitisation [45].

IgE-mediated reactions to fish are the end-result of a process that, such as every allergic reaction, starts with the absorption of antigens through the intestinal epithelium (ingestion), lung mucosa (inhalation), or the skin (contact). The latter can happen via either a passive system (paracellular diffusion) or an active system, which involves goblet cells or dendritic cells [46,47]. After absorption, dendritic cells and macrophages usually participate in the tolerance mechanisms in which regulatory T cells are produced, which suppress Th2 response, preventing IgE sensitisation development [48,49]. In allergic patients, though, this process fails, and antigen-presenting cells switch to a Th2 response, in which one of the leading players is IL-4. This cytokine induces the development of Th2 cells specific for the presented antigen [50]. Thus, tolerance breaks down, and interaction between Th2 and B cells leads to the production of antigen-specific IgE, which binds to IgE surface receptors of mast cells/basophils. Re-exposure to the antigen and binding and cross-linking allergen-specific IgE on mast cells and basophils leads to degranulation, with the release of preformed granules containing histamine and tryptase, and the new production of mediators such as prostaglandins and leukotrienes [51]. Those mediators’ effects include vasodilatation, mucous secretion, smooth muscle contraction, and chemotaxis of other inflammatory cells, which maintain and amplify the inflammatory process, leading to typical clinical allergic reaction manifestations [52].

Fish allergy in children can manifest after either ingestion, skin contact, or inhalation of the antigen. The most common route of sensitisation, especially in children, is the digestive system: fish antigens take only around 10 minutes to be absorbed after ingestion, so even a partial impairment of the denaturing effect of gastric acid (such as in patients using antiacid medications) can lead to partial digestion and increased intake of antigenic peptides [53,54]. An increase in gastric pH level from 2 to 3 caused a 10–30-fold increase in allergenicity of codfish allergens [54]. IgE-mediated signs and symptoms after oral ingestion are usually rapid in onset, especially true for fish, considering its absorption rate.

Classically, those clinical manifestations are incredibly varied, both in nature and severity. They range from mild oral allergy syndrome and the most common urticaria/angioedema, reported by up to 70% of fish-allergic patients in the US [55], to life-threatening events like anaphylaxis. Among the patients with food-induced fatal anaphylaxis, 1 of 32 deaths was caused by fish, and in a report of seven deaths, one was attributed to crab and one to fish [55]. Interestingly, fish represented the third most common cause of anaphylaxis in a cohort of Portuguese children, in which it accounted for 18.8% of cases of anaphylaxis [56]. However, there is significant variability in the prevalence of anaphylaxis caused by fish between different countries. For example, fish was a causative agent in 2.1% of Korean children with anaphylaxis [57]. Another common presentation of fish allergy is gastrointestinal involvement, with clinical manifestations like nausea, vomiting, and diarrhoea [58]. Additionally, food allergy can induce exercise-induced anaphylaxis, in which signs and symptoms appear only if the subject performs physical activity around few hours from ingesting the food towards which they are sensitised [59,60,61]. Indeed, food-related exercise-induced anaphylaxis cases have been described for fish [62,63].

Bronchospasm and asthma have been reported after ingestion of fish, but their occurrence is also possible after the inhalation of fish allergens. The latter route of exposure is recurrent in seafood industry workers and handlers [64,65,66,67,68], but children can easily be exposed to aerosolised proteins generated in the cooking process [69,70]. Cooking is an especially pernicious process when dealing with fish allergy, as it may increase the allergenicity of fish allergens in some cases [71,72]. Van der Ventel found out that mice exposed to cooked pilchard developed higher IgE levels when compared to raw extract, despite manifesting a narrower range of antigen sensitisation, showing specific IgE almost exclusively directed towards parvalbumin, which is thermally stable. The explanation for this phenomenon is that, in cooked fish, parvalbumin concentration increases, while the minor allergens’ concentration decreases, being more easily denatured. This also contributes to a potential explanation of why parvalbumin is the most prevalent antigen responsible for fish allergy since fish is predominantly consumed cooked [72]. The main presentation of fish allergy caused by sensitisation by inhalation of antigens is, as previously reported, the appearance of bronchospasm or other upper- and lower-respiratory tract clinical manifestations [64,65,66,67,68] when re-exposed to the same inhaled allergen. However, urticaria, conjunctivitis, and even anaphylaxis have also been reported [73]. In some cases, fish has been reported to be tolerated when eaten and elicited signs and symptoms only when allergens were aerosolised via cooking [74], in line with the previously mentioned allergenicity changes of cooked fish [71,72].

The final sensitisation route is direct skin contact with fish protein, resulting in contact urticaria and eczema or anaphylaxis in rare cases [65,75]. The latter method of sensitisation is mainly reported in workers from the seafood-processing industry, fishers, and professional cooks, but it has also been reported in paediatric age patients [67,76]. The presence of an impaired skin barrier function is a significant risk factor for fish sensitisation: this is also confirmed by the fact that patients with filaggrin (a protein essential for the maintenance of normal epidermal homeostasis) loss-of-function gene mutations have been shown to have a 4-fold risk of developing fish allergy when compared with nonmutated controls [77].

## 4. Fish Allergens and Cross-Reactivity

**Figure 1:**
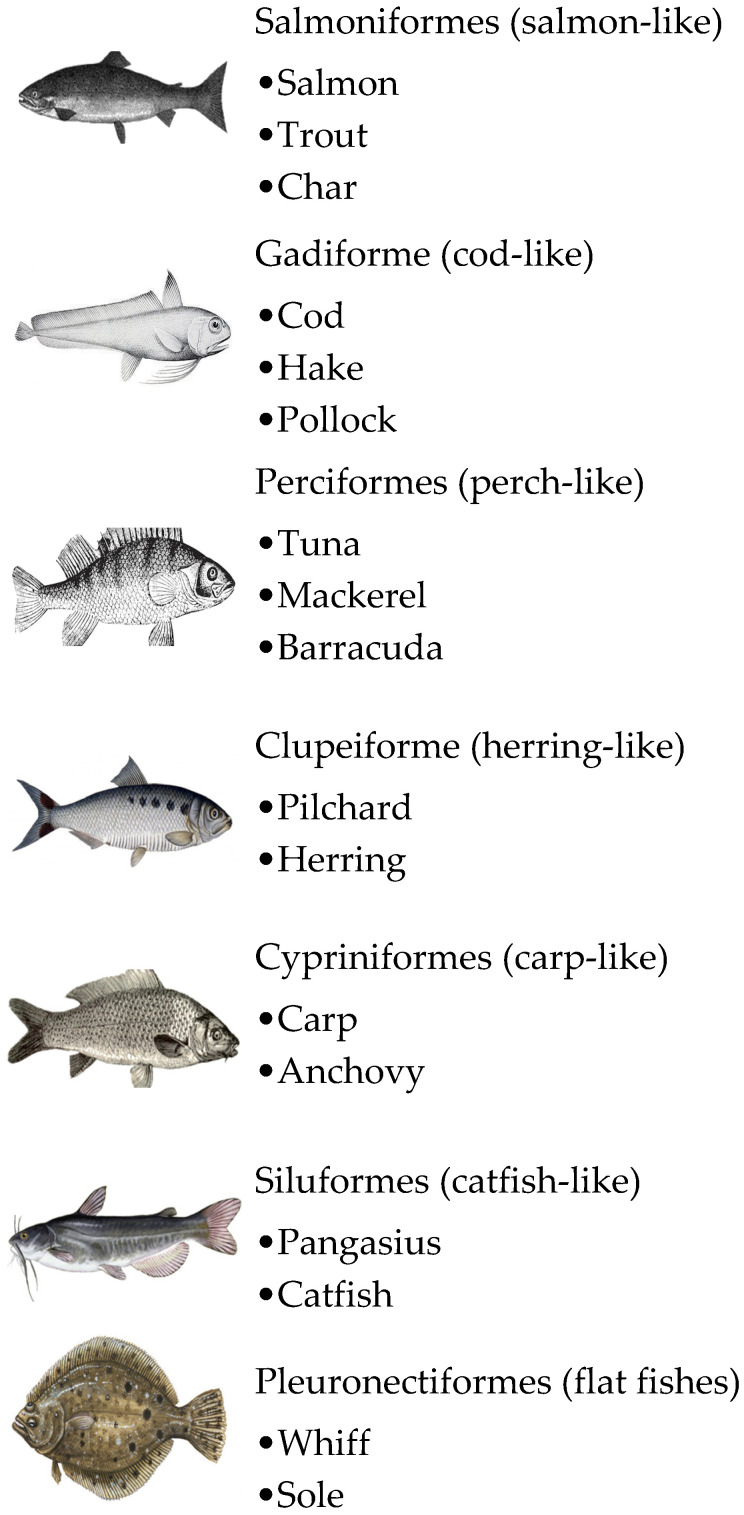
Taxonomic definition of the most commonly consumed fishes.

Fish are mainly categorised into two classes as bony fish (*Osteichthyes*) and cartilaginous fish (*Chondrichthyes*) [58]. Most of the edible fish are bony fish, while rays and sharks belong to the cartilaginous group. Although there is vast biodiversity among fishes (more than 32,400 species), most of the bony fish belong to a limited number of orders, the cod-like (*Gadiformes*), salmonlike (*Salmoniformes*), perchlike (*Perciformes*), herringlike (*Clupeiformes*), carplike (*Cypriniformes*), catfishlike (*Siluriformes*), and flatfishes (*Pleuronectiformes*) (Figure 1) [78]. Identification of fish allergens from different regions and species facilitates the diagnosis and treatment of fish allergy. So far, a limited number of species have been analysed.

### 4.1. Parvalbumin

Parvalbumin was first detected as a fish allergen in Baltic cod (Gad c 1 or Allergen M) in 1969 [79]. This protein’s allergenicity was demonstrated in other fish species such as carp, salmon, pilchard, tuna, and mackerel [71,80,81,82]. Parvalbumin is a calcium-binding protein with a molecular mass of about 12 kDa, which participates in muscle fibre relaxation and highly resistant to heat and enzymatic digestion [58]. Parvalbumin represents the major cross-reactive allergen in fish allergy [78].

Based on amino acid sequences, two distinct isoform lineages have been identified as alpha and beta parvalbumin. Fish species may have alpha and beta, but most of the allergenic parvalbumins belong to the beta lineage, found in bony fish [83]. Alpha parvalbumin is mostly found in the muscle of cartilaginous fish and seems to be nonallergenic. The sequence identities have been reported for beta parvalbumins to vary significantly between 46% and 99%, possibly explaining monosensitivity to specific fish species [84]. A recent study by Kalic et al. demonstrated with a food challenge that patients with allergy to bony fish can consume ingestion of ray, another type of cartilaginous fish [85].

White muscles of fish contain a higher concentration of parvalbumin than the dark muscles. Hence fish with white muscle such as haddock and cod seem to be more allergenic than fish with more dark muscles such as mackerel, tuna, and swordfish [86]. Indeed, parvalbumin content differs in fish species; swordfish and tuna contain <1 mg of parvalbumin per gram of fresh fillet, while the concentration of parvalbumin is higher than 2.5 mg per gram in cod and carp [87]. Allergenic variability among the fishes may help the patients to tolerate fish species with low parvalbumin content, although they have allergic reactions with fishes containing high parvalbumin content.

### 4.2. Enolase and Aldolase

In addition to parvalbumin, aldolase A (40 kDa) in Pacific salmon and beta enolase (50 kDa) in bream were also detected as fish allergens [88,89]. These allergens are abundant in fish muscle and play a role in glucose metabolism. Parvalbumin sensitised patients may also have IgE reactivity to enolase and aldolase. Kuehn et al. reported a group of patients who were not sensitised to parvalbumin but had IgE reactivity to enolase and aldolase from salmon, tuna, and cod [90]. IgE to enolase and aldolase were found in 62.9% and 50% of the patients, respectively. The authors described IgE to enolase and aldolase as clinically relevant, particularly when sensitisation to parvalbumin was not detected, and this might be related to enolase and aldolase being not heat stable. By using IgE-inhibition enzyme-linked immunosorbent assay (ELISA), limited interspecies cross-reactivity was detected for aldolases and enolases [90].

### 4.3. Collagen

Type I collagen was detected as a second fish allergen in 2000 [91]. In Japan, IgE reactivity to fish collagen was observed in 50% of the patients with fish allergy [92]. The same group also reported that sera of patients retained IgE reactivity to fish collagen after heated at 100 °C for 320 min and 140 °C for 10 min [93]. A study on 100 Australian fish allergic children demonstrated that 21% were sensitised to collagen from salmon, tuna, and Asian seabass, and basophil activation was demonstrated [85]. The authors suggest to include this heat-stable allergen fish allergy diagnosis.

On the other hand, Hansen et al. investigated the allergenicity of gelatine in 30 fish allergic patients based on the results of double-blind placebo-controlled food challenges (DBPCFCs). They reported that 90% of fish-allergic individuals with 95% certainty would not have shown a reaction to the ingestion of a 3.61 g cumulative dose of fish gelatine, which questions the clinical relevance of gelatine in fish allergy [94].

### 4.4. Other Allergens

In 2013, a muscle protein tropomyosin, a pan-allergen for shellfish, was identified as a fish allergen in patients with tilapia sensitisation [95]. A recent study from Australia among 77 paediatric patients with confirmed fish allergy demonstrated that up to 32% were sensitised to tropomyosin from salmon or Asian seabass [96]. Furthermore, a fish yolk protein vitellogenin in Beluga caviar and several molecules such as aldehyde phosphate isomerase, triose-phosphate isomerase, glyceraldehyde-3-phosphate dehydrogenase, and creatine kinase have been displayed as potential allergens in different species of fish [72,97,98]. However, the clinical relevance of these allergens requires further investigation.

A summary list of fish allergens officially recognised by the World Health Organization/International Union of Immunological Societies (WHO/IUIS) is reported in Table 1.

### 4.5. Cross-Reactivity

Clinical cross-reactivity has been shown among the fishes even from taxonomically distinct families. This event has been explained by IgE reactivity to parvalbumin, the major fish allergen, which is responsible for the clinical manifestations in 90% of the patients [78]. Van Do et al. investigated 10 patients by using SPT, sIgE, and immunoblotting, and reported that cod (Gad c 1), pollack (The c 1), salmon (Sal s 1), wolfish, and herring were the most cross-reacting allergens, while mackerel, tuna, halibut, and flounder were found as the least allergenic [110]. In Australia, a study among children, with confirmed fish allergy demonstrated different sensitisation among 12 different species with catfish being the most allergenic fish when combining IgE reactivity to several major allergens [112].

On the other side, selective allergy to one type of fish species have also been demonstrated in salmonids where patients had allergic signs and symptoms following trout or salmon consumption, but not reactive to cod, herring, carp, redfish [113,114]. In Italy, monosensitisation to tropical sole was reported in a patient who did not react to cod, salmon, lemon sole, tuna, and swordfish [115]. Although eliminating all the fish species due to cross-reactivity in patients with fish allergy in previous years is common, these findings suggest that some patients can consume some fish species. However, it would be appropriate to confirm tolerance to safe alternatives by food challenge tests before introducing them into patients’ diets.

For the fish allergic patients, the possibility of cross-reactivity to other fish species is about 50%, which is lower than the cross-reactivity between shellfish species reported as 75% [116,117]. Currently, cross-reactivity between fish and shellfish has been shown in a few studies [118,119]. Moreover, cross-reaction between frog and fish beta parvalbumin has been described in a study, including 15 patients [120]. Cross-reactivity between fish and other vertebrate meats has been reported, e.g., between fish and chicken meat involving parvalbumin, enolase, and aldolases, named “fish-chicken syndrome” [121]. A study among 66 fish allergic individuals demonstrated SPT reactivity in 60% to crocodile meat, with cross-reactive parvalbumin demonstrated to be the major allergen [122].

Finally, fish tropomyosin’s cross-reactivity with shellfish and clam was investigated with a nonthermal extraction technique [123]. Although a high sequence similarity was demonstrated, fish tropomyosin did not show cross-reactivity with shrimp and clam tropomyosin. In another study of the same research group, B cell epitopes from shrimp were reported to have a high cross-reactivity with clam tropomyosin (>80%) and low cross-reactivity with fish tropomyosin (<20%) [124]. These two studies emphasise the need for further investigations on this subject.

### 4.6. Food Processing

Food processing may alter the allergenicity of fish species. For example, the palvalbumin allergenicity is low in canned fish and can be tolerated by some patients who cannot consume fish in fresh form (Table 2). On the other hand, heating is shown to increase the allergenicity of fish, as is the case for peanut allergens. Heat resistance is considered lower in enolase and aldolases compared to parvalbumin [90]. Furthermore, the detectability of fish allergens in processed food might be altered, as demonstrated by a recent study. Ruthers et al. compared three commercial ELISA tests for fish allergens and demonstrated that only 26–61% of fish extracts of the 57 fish species were detected, while none of the nine cartilaginous fish were detected [125].

## 5. Diagnosis

Confirming IgE-mediated fish allergy diagnosis is essential in terms of avoiding both “over”- and “under”-diagnosis. In clinical practice, the diagnosis is commonly based on a convincing history and demonstrating the presence of sensitisation via in vivo (SPT) or in vitro (sIgE) tests [126]. However, specific IgE positivity may merely reflect sensitisation, but not necessarily clinical reactivity. In other words, some patients who present sensitisation to the fish allergen, particularly with low levels of sIgE, can tolerate ingestion of certain fish species. Therefore, oral food challenges (OFC) remain the gold standard test for food allergy diagnosis.

### 5.1. Clinical History

A detailed clinical history is the mainstay of the diagnosis. Revealing a temporal association between fish consumption and allergic clinical manifestations will point out food allergy as the source of the issue. More information including signs, symptoms, and severity along with the type of the suspected fish, provoking quantity, the time interval between ingestion and onset of clinical manifestations, triggering factors (e.g., exercise, illness, drugs), having any previous reaction with the same or different kind of fish is queried during the assessment. Testing for the fish, which is currently tolerated, is not required. Conditions such as scombroid poisoning, allergy to fish parasite Anisakis, and allergy to additives in canned fish should be considered to avoid misinterpreting fish allergy. Following the medical history, evaluation of SPTs, sIgEs, and performing OFCs are recommended before establishing allergy diagnosis.

### 5.2. SPTs

Since the first description by Lewis and Grant in 1924 [127], SPT has been commonly used to diagnose of IgE-mediated allergic diseases due to its properties as sensitive, easily applicable, cost-effective, and giving rapid results. The immunologic mechanism involves releasing histamine and other mediators from mast cells following cross-linking of specific IgE with allergens applied to the skin [128]. The test is performed by applying a drop of fish allergen solution on the patient’s upper back or forearm’s volar surface and pricking it through a lancet or another commercial test device. Placing allergens 2 cm or more apart is appropriate for preventing false-positive results. The back of the patients is more reactive than the forearm, resulting in larger wheal sizes. Histamine (10 mg/mL) and normal saline are used as positive and negative controls, respectively [129]. A positive reaction is defined as the diameter of wheal size equal to or more than 3 mm with a negative control read after 15–20 min.

SPTs are not recommended in patients with severe dermographism, uncontrolled atopic dermatitis, or asthma, and in patients who use drugs that may interfere with skin reactivity (e.g., antihistamines, tricyclic antidepressants) [130]. The tests can be performed using commercial fish extracts or prick-by-prick tests with fresh food for the fish allergens that are not available in the market due to the number of fish species. Although it is considered a safe procedure, several anaphylactic reactions following SPTs with fish allergens have been reported [131,132]. A 4-year old girl without any history of a systemic reaction has been reported to experience anaphylaxis during the prick-to-prick tests with eight fish species [133]. Therefore, well-trained personnel should perform the tests in an adequate-equipped setting to treat potential severe reactions.

Given that some of the patients may be sensitised to one or a few fishes, testing with multiple fish species may help find safe alternative fishes and manage treatment plans. Many factors such as technique, the skill of the testing staff, the test instrument, potency, and stability of test reagents, preservatives in the extract may affect the results of SPTs. Ruethers et al. investigated 26 commercial fish extracts from five different companies, strikingly finding more than 10-fold variation in protein content, allergen concentration, and IgE reactivity using immunoblotting and mass spectrometry assays [134]. Using recombinant proteins that can be standardised in their quantity and quality may be a potential option to improve the process. However, excluding other potentially essential allergens from natural extracts is a handicap of this approach [135]. Van do et al. investigated the reactivity of SPT of natural and recombinant parvalbumins from cod, pollock, and salmon. However, the results yielded inadequate responses with recombinant versions, presumably stem from the specific conformational binding of high-affinity IgE binding motifs [110]. Although SPTs have high sensitivity and negative predictive value (NPV), they are not specific, and positive predictive value (PPV) is rarely higher than 50% [136].

Moreover, the canning process may alter the allergenicity of protein fractions and lower IgE specific binding to the fish extracts in canned form compared to raw and cooked extracts was shown by immunoblot analyses. In a group of children with fish allergy, 20% of the children allergic to salmon or tuna could tolerate the fish in a canned form, which consumption was associated with a decrease in SPT wheal sizes, addressing that ingestion of canned fish may have led to an induction of tolerance in these children [137]. Collagen allergy is also another critical issue in the diagnosis of fish allergy. As collagen is water-soluble when heated, Chikazawa et al. suggested performing prick-by-prick tests with heated fish following a negative test with raw fish to overcome the obstacle of overlooking allergy to collagen [138]. Subsequent studies by Kalic et al. demonstrated that collagen is only affectively extracted at very acidic conditions [85].

### 5.3. sIgEs

Specific IgEs can be measured with different diagnostic systems, whose results are not interchangeable [139]. Currently, the ImmunoCAP (Phadia/Thermo Fisher Scientific, Uppsala, Sweden) is more frequently used, with 28 fish extracts available for measuring fish allergens. For predicting clinical reactivity, cut-off points of specific IgE are determined for some foods, e.g., milk, egg, peanut, tree nuts in different paediatric populations [140,141,142]. For fish allergy, specifically for cod allergy, Sampson et al. found that a serum-specific IgE level of 20 kU/L can predict a positive reaction with 95% certainty in children [143]. However, it is questionable whether the levels can be extrapolated to other fish species allergy diagnosis and other populations. Notably, very low food sIgE levels may be associated with positive reactions in 10–25% of patients [144]. Beale et al. reported anaphylactic reactions to pilchard and anchovy in patients with IgE levels as low as 1 kU/L [71]. A retrospective chart review of adults and children with seafood allergy who underwent open OFCs between 2008 through 2019 in the US revealed a significant difference between fish sIgE values for negative (<0.34 kUA/L) and equivocal (<0.34 kUA/L) OFCs versus positive (1.63 kUA/L) OFCs (*p* = 0.023) but not for shellfish (*p* = 0.272) [145]. Logistic regression analysis determined a cut-off specific IgE level of 1.99 kUA/L with an 85% negative challenge rate, and the NPV was 82.35%. In a North European study, 35 patients with fish allergy, aged 5–19, were evaluated by DBPCFCs. Among the 24 clinically reactive participants, cod specific IgE was above 8.2 kU/L in 19 patients, and salmon sIgE was above 5.0 kU/L in 20 patients [146]. The authors suggested that these cut-off levels might help reduce the number of food challenge tests.

### 5.4. OFCs

Oral food challenges are still the most accurate way of detecting clinical allergy. However, these tests are time-consuming, labour-intensive, and expensive.

**Figure 2:**
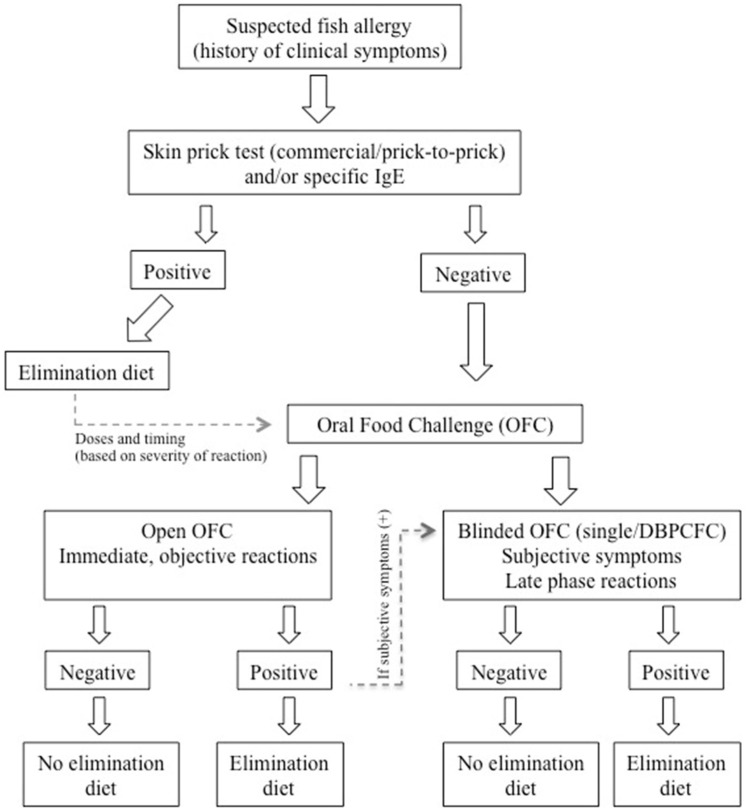
Algorithm modified from the article of Niggemann et al. for the diagnosis of IgE mediated fish allergy in children [147] (DBPCFC: double-blind placebo-controlled food challenge test).

The tests can be performed in three forms:

- Open OFC: the food is administered in its daily-consumed form.
- Single-blind placebo-controlled food challenge (SBPCFC): the patient is blinded to the tested food.
- DBPCFC: both patient and observing heath care staff are blinded to the tested food.
- In allergy practice, open food challenges are usually preferred, particularly in patients with clear history. However, awareness of the tested food by both the patient and the physician may bias false-positive results, particularly in patients with subjective symptoms such as abdominal pain. In these patients, DBPCFCs help confirm or rule out the disease. An algorithm for the diagnosis of fish allergy is indicated in Figure 2 [147].

In food challenges, suspected fish is given in gradually increasing doses until an age-appropriate serving is reached. A challenge can be ceased at the first clinical manifestations of an allergic reaction, preventing more severe reactions, as well as determining the eliciting threshold dose. In general, 5 mg is the proposed starting dose for fish; however, the dose will be less in patients with a severe reaction history, according to the physician’s decision [148]. Some studies suggested 3 mcg of food protein as the first dose to determine no observed adverse effect [149,150]. Ballmer-Weber et al. evaluated patients with food allergies who underwent DBPCFCs for identifying the threshold dose distributions in the European population. For fish, estimated doses eliciting reactions in 10% of the allergic population (ED10) were 27.3 mg of protein, higher than a hazelnut, peanut, and celery ranging from 1.6 to 10.1 mg of protein [151]. In SBPCFCs and DBPCFCs, fish is blinded by masking it with other foods tolerated by the patient. The active and placebo foods will be served on separate days or, if it is preferred to be given on the same day, there should be 2–3 h intervals between the tests [152,153]. Open OFCs may also be performed in situations where masking is tricky due to the high total cumulative dose of fish. For the Europrevall study conducted in 12 different allergy clinics across Europe, DBPCFCs were standardised for an extensive evaluation of food allergy. For fish, a maximum of 1 g of protein, which is equal to 5 g cooked fish, was blinded in a chocolate matrix, and the doses were given as 3 mcg, 60 mcg, 600 mcg, 12 mg, 120 mg, and 1 g fish protein [154]. If there was no reaction, open challenges with cooked Atlantic cod 2, 6, and 12 g protein in three doses were performed.

Additionally, Vassilopoulou et al. developed and validated a recipe including chicken breast, potato powder, salt, pepper, spearmint, dill, and vinegar for blinding fish in DBPCFC tests [155]. The recipe was also found acceptable by the patients regarding appearance, blinding, taste, and odour. Although DBPCFC tests are the gold standard tests for the diagnosis, anaphylaxis may occur even with low SPT and sIgE levels. Hence, OFC’s decision is challenging for physicians, and benefits and costs should be considered.

### 5.5. Component Resolved Diagnosis (CRD)

The low specificity of SPT and specific IgE levels and OFC safety concerns have led researchers to develop a new diagnostic test to overcome these methods’ drawbacks. CRD measures IgE antibodies to individual allergen components and gives more information on cross-reactivity and risk for severe reactions. Recent studies found Ara h 2 and Cor a 14 as biomarkers for severe peanut and hazelnut reactions, respectively [142,156]. The clinical utility of CRD has not been fully demonstrated in fish allergy yet.

Fish parvalbumins rGad c 1 and rCyp c 1 can be measured by the ImmunoCAP system (Phadia/Thermo Fisher Scientific, Uppsala, Sweden). Another technique Microarray-based ImmunoCAP ISAC system (Immuno-Solid Phase Allergen Chip; Phadia/Thermo Fisher Scientific, Uppsala, Sweden), can detect IgE reactivity to 112 inhalation and common food allergens that includes cod (rGad c 1) allergens [135]. Although the ImmunoCAP and ISAC assays’ outcomes are comparable, the sensitivity of ImmunoCAP is higher than ISAC. On the downside, it requires a larger amount of sera because it analyses each allergen component individually [157]. In a retrospective study, baseline SPT results, sIgE and rGad c 1 levels of 81 patients with fish allergy were compared to 60 patients who acquired tolerance to at least one fish species [158]. The authors found that the decrease in the mean SPT wheal size and specific IgE to rGad c 1 for salmon and hake can be useful markers to evaluate the development of tolerance in fish allergy.

Recent studies investigated recombinant fish parvalbumin for clinical cross-reactivity in children with fish allergy [90,159]. On the other hand, some patients may present with monosensitisation to some fish species. Kelso et al. reported allergy only to swordfish while the tests to other nine commercial fish extracts were negative [160]. Another study found a correlation with fish enolases and aldolases specific IgE and clinical sensitivity in three cod sensitised patients who were not allergic to parvalbumin [161]. A testing panel including minor fish allergens such as enolases, aldolases, and perhaps collagen could be of added value for the component-resolved fish allergy diagnosis. Overall, while CRD provides more information and improves the diagnostic tests, it does not eliminate the need for OFC in many patients.

### 5.6. Basophil Activation Test (BAT)

BAT has been investigated as a novel tool in food allergies to provide a biomarker for predicting clinical reactivity, reaction severity, and to reduce the need for OFCs [162]. The BAT utilises flow cytometry to determine the expression of activation markers (e.g., CD 63, SD203c) on the surface of basophils following the cross-linking of high-affinity IgE receptor (FcεRI)-bound IgE antibodies that arise from allergen or anti-IgE stimulation [163]. In 2015, 67 patients aged 12–45 years old underwent DBPCFC tests with peanut, hazelnut tree nut, fish, shrimp, and sesame to determine whether SPT, sIgE, allergen-specific IgG4, component testing, and basophil activation are associated with DBPCFCs results and the severity of allergic reactions during challenge [164]. The results revealed that BAT could discriminate reactive from nonreactive patients, and it was positively correlated with DBPCFC severity scores. Based on the receiver operating characteristic (ROC) curve analysis, basophil reactivity had the largest area under the curve (AUC) at 0.904, and sIgE had an AUC of 0.870. Dose-dependent outcomes with BAT were addressed, as stimulation with 200 ng/mL allergen showed the highest overall accuracy and best diagnostic performance [164]. A recent study investigated 51 Japanese children with fish allergy who underwent BAT using in-house fish extracts of 15 different kinds of fish species [165]. The AUC was high (0.72–0.88) for the five most commonly consumed fish species (salmon, mackerel, tuna, red sea bream, and yellowtail), and the diagnostic accuracy was between 0.74 and 0.86. The diagnostic performance of BAT was found similar to sIgE. However, the authors emphasised BAT’s usefulness, particularly for fish species with no available sIgE test [165].

Although there are promising results regarding its value in clinical reactivity and severity of allergic reactions, BAT is commonly used for research purposes and not yet implemented in daily practice, most probably due to technical difficulties and high cost.

## 6. Management

Today, the only widely accessible and clinically proven approach to fish allergy is food avoidance and rescue medication (e.g., adrenaline, corticosteroids, antihistamines) in accidental exposure [126]. Food avoidance poses many problems and is not easily maintained. Since the primary fish allergen, parvalbumin, is highly conserved between fish species, cross-reactivity is expected, so it is often insufficient to avoid only the original offending fish [90,166,167,168]. Given this, mono- and oligo-sensitisation to one or few fish species have been documented in adults and children [161,169,170,171], and often ingestion of fish with a higher content of red meat, such as tuna, are tolerated even in polysensitised patients [110,172], probably due to the lower concentration of parvalbumin found in red muscle [80]. Different processing methods have different effects on allergenicity: canning, marinating, or fermenting may result in tolerance of previously nontolerated raw fish [137,173,174]. On the other hand, the heating process is not enough to destroy the fish’s main allergenic proteins and will likely trigger sensitised patient’s reactions [71]. The extent of fish elimination should thus be ideally tailored per patient.

Cross-reactions are not relegated to other fishes: cross-reactivity with shellfish is rare but has been reported for fishes like tilapia [119]; cross-reactivity has also been documented for chicken [121,175] and crocodile meat [176]. Hidden fish allergens in food represent another sizable threat to fish-allergic patients. In a study by Anibarro et al., 35% of fish allergic patients reacted to fish proteins hidden in other foods [177]. In addition not all allergens from different fish species are detected by commercial tests [125].

Food is also not the only source of fish allergens, hidden or not, and fish elimination diets are not effective when dealing with these sources: aerosolisation of fish proteins when cooking, as mentioned above, is a common source of highly allergenic parvalbumin [70,72,73]. A study performed in Norwegian homes showed fish allergens in 46% of mattresses in patients’ bedrooms [178]. Additionally, to the difficulty and fallibility of even a correctly performed fish elimination diet, the fact itself of eliminating food from the diet has social and psychological implications: feeling different because of the diet, worrying about foods, increased anxiety and stress in both the children and their families, increased feeling of responsibility, adverse effect on social activities. Children with food allergies experience food-related bullying because of their diet [179,180,181,182]. Considering this, it is easy to understand why the attention has shifted towards the development of specific immunotherapy: the first documented trial of fish immunotherapy dates back to 1930, with Freeman’s “Rush inoculation” [183]. Multiple desensitisation approaches have been tried over the years. In their most recent endeavours, Patriarca et al. [184,185] attempted oral desensitisation with boiled cod. However, several allergic reactions during OIT and the following observation period were documented both in adults and children, and they still did not reach a tolerated dose comparable to a typical fish portion. Recently, similar approaches were performed by D’Amelio in 2017 and Martore-Calatayud in 2019 by using hake instead in paediatric populations, with promising results [186,187].

The deepening of molecular knowledge about the fish allergen parvalbumin has allowed the development of hypoallergenic recombinant parvalbumin with preserved immunogenicity in recent years. Allergenicity is determined by conformational epitopes of parvalbumin [188], meaning that conformational changes can create proteins deprived of IgE-binding epitopes but can still determine an IgG response. The previous has been demonstrated with carp parvalbumin [72,189], and it represents a promising route for the development of fish allergy vaccines [99] and subcutaneous specific immunotherapy. The first-in-man trial has been performed in 2013 in Denmark, and it was followed in 2015 by a phase 2b clinical trial in six countries [190]. Recently, passive immunisation’s effectiveness via blocking antibodies created with hypoallergenic recombinant parvalbumin has been trialled in murine models of fish allergy [191].

## 7. Conclusions

Fish allergy in children is a growing health concern, and the prevalence has been reported between 0% and 7% worldwide. The reactions can occur after ingestion, skin contact, or inhalation of the antigen and range from mild symptoms to life-threatening anaphylaxis [192]. Although a limited number of species have been analysed, parvalbumin, aldolase A, beta enolase, tropomyosin, collagen, vitellogenin have been identified as fish allergens. Parvalbumin, the major allergen, is responsible for the allergic reactions in the vast majority of the patients. Skin prick tests and specific IgE are most commonly used tests. However, DBPCFC tests remain the gold standard tests for the diagnosis of fish allergy. Recent studies investigated CRD and BAT as a novel tool for predicting clinical reactivity, reducing the need for OFCs, and yielding favourable outcomes. Currently, the use of rescue medication (e.g., adrenaline, corticosteroids, antihistamines) in accidental exposures is the only clinically proven and widely accessible approach to fish allergy. New promising approaches via the modulation of the pathological immune response in fish allergy are under investigation and may lead to novel therapeutic options in the future.

## Figures and Tables

**Table 1:** Summary of the fish allergens based on the search in the World Health Organization/International Union of Immunological Societies (WHO/IUIS) database (www.allergenonline.org).

Fish Species	Common Name	Nomenclature	Order	Allergen	MW * (kDa)	Ref
*Clupea harengus*	Atlantic herring	Clu h 1	Clupeiformes	β-parvalbumin	12	[99]
*Cyprinus carpio*	Common carp	Cyp c 1	Cypriniformes	β-parvalbumin	12	[100]
*Gadus callarias*	Baltic cod	Gad c 1	Gadiformes	β-parvalbumin	12	[101]
*Gadus morhua*	Atlantic cod	Gad m 1	Gadiformes	β-parvalbumin	12	[102]
	Atlantic cod	Gad m 2	Gadiformes	β-enolase	47.3	[90]
	Atlantic cod	Gad m 3	Gadiformes	Aldolase A	40	[90]
*Lates calcarifer*	Barramundi/Asian Seabass	Lat c 1	Perciformes	β-parvalbumin	11.5	[103]
	Barramundi/Asian Seabass	Lat c 6	Perciformes	Collagen alpha	130–140	[104]
*Lepidorhombus whiffiagonis*	Megrim, whiff, turbot fish	Lep w 1	Pleuronectiformes	β-parvalbumin	11.5	[105]
*Oncorhynchus mykiss*	Rainbow trout	Onc m 1	Salmoniformes	β-parvalbumin	12	[106]
*Rastrelliger kanagurta*	Indian mackerel	Ras k 1	Scombriformes	β-parvalbumin	11.3	[107]
*Salmo salar*	Atlantic somon	Sal s 1	Salmoniformes	β-parvalbumin	12	[108]
	Atlantic somon	Sal s 2	Salmoniformes	β-enolase	47.3	[90]
	Atlantic somon	Sal s 3	Salmoniformes	Aldolase A	40	[90]
*Sardinops sagax*	Pacific pilchard	Sar sa 1	Clupeiformes	β-parvalbumin	12	[71]
*Sebastes marinus*	Ocean perch, redfish	Seb m 1	Scorpaeniformes	β-parvalbumin	11	[109]
*Thunnus albacares*	Yellowfin tuna	Thu a 1	Perciformes	β-parvalbumin	11	[110]
	Yellowfin tuna	Thu a 2	Perciformes	β-enolase	50	[90]
	Yellowfin tuna	Thu a 3	Perciformes	Aldolase A	40	[90]
*Xiphias gladius*	Swordfish	Xip g 1	Perciformes	β-parvalbumin	11.5	[105]
*Oreochromis mossambicus*	Mozambique tilapia	Ore m 4	Perciformes	Tropomyosin	33	[95]
*Oncorhynchus keta*	Chum salmon	Onc k 5	Salmoniformes	Vitellogenin	18	[111]

* Molecular weight.

**Table 2:** Food processing effects in parvalbumin content (ELISA) modified from the article of Kuehn et al. [126].

Type of Fish	Presentation	Parvalbumin Content (mg/g)
Cod	Raw	1.5–2.5
	Smoked	1.0–1.3
	Cooked	1.3–1.9
Salmon	Raw	1.9–2.5
	Smoked	0.7–1.0
	Cooked	1.5–1.9
Carp	Raw	2.5–5.0
	Cooked	2.1–4.0
Tuna (white muscle)	Raw	0.01–0.05
	Cooked	0.01–0.03
Tuna (dark muscle)	Raw	ND
Tuna	Canned	ND

ND: non detectable.

## Data Availability

All data presented in this review are included in this article.
